# Harnessing biogenic nanoparticles for combating antibiotic resistance: green synthesis, mechanistic insights, and biotechnological applications

**DOI:** 10.3389/fbioe.2026.1752199

**Published:** 2026-03-26

**Authors:** Emad M. Abdallah, Abdulrahman Mohammed Alhudhaibi, Ibrahim Mohammed Hussaini, Asmau Nna Sulaiman

**Affiliations:** 1 Department of Biology, College of Science, Qassim University, Buraydah, Saudi Arabia; 2 Department of Biology, College of Science, Imam Mohammad Ibn Saud Islamic University (IMSIU), Riyadh, Saudi Arabia; 3 Department of Microbiology, Faculty of Life Sciences, Ahmadu Bello University Zaria, Zaria, Nigeria

**Keywords:** antimicrobial resistance, biomedical applications, drug discovery, green synthesis, mechanistic insights, microorganisms, nanotechnology, phytochemicals

## Abstract

Antimicrobial resistance (AMR) continues to escalate worldwide, reducing the effectiveness of existing antibiotics and leading to an urgent need for alternative antimicrobial strategies. Biogenic nanoparticles (BNPs) are nanomaterials synthesized using plants, microorganisms, algae, or isolated biomolecules through biologically mediated processes. These green synthesis routes typically produce nanoparticles that are naturally capped with biological compounds such as phytochemicals, proteins, or polysaccharides, which can influence particle stability, surface chemistry, and biological interactions. The current review summarizes recent advances in the biosynthesis of BNPs, focusing on how biological metabolites regulate metal ion reduction, nucleation, growth, and stabilization, thereby shaping nanoparticle size, morphology, and antimicrobial behavior. BNPs including silver, gold, copper oxide, zinc oxide, iron oxide, titanium dioxide, and selenium have been widely investigated for antibacterial activity. Experimental studies indicate that these materials can act through multiple mechanisms, such as disruption of bacterial membranes, generation of reactive oxygen species, interference with metabolic processes, damage to DNA and proteins, inhibition of quorum sensing, and suppression of biofilm formation. Reported activity spans several WHO-priority and ESKAPE pathogens, largely based on *in vitro* studies and a limited number of preclinical *in vivo* models. BNPs have also been explored as adjunct platforms in combination with antibiotics, essential oils, or phytochemicals, where synergistic effects may reduce required drug concentrations and improve activity against resistant strains. In parallel, their physicochemical tunability has supported experimental applications in wound-related systems, antimicrobial coatings, drug delivery research, biosensing, and food-packaging materials. Despite these encouraging findings, major challenges remain. Variability in green synthesis protocols leads to inconsistent physicochemical properties, scalability remains limited, and comprehensive long-term toxicological, pharmacokinetic, and environmental safety data are still lacking. Addressing these issues is essential before BNP-based strategies can progress toward regulatory evaluation and clinical use. In conclusion, biogenic nanoparticles represent a promising but still experimental platform that may contribute to future antimicrobial development if supported by standardized synthesis, rigorous safety assessment, and translational research.

## 1 Introduction

Antibiotic resistance continues to rise at an alarming pace, steadily narrowing available treatment options and underscoring the urgent need for unconventional antimicrobial solutions. We have now reached a critical point where many experts consider that we are entering a real post-antibiotic era (Abdallah et al., 2023). Antimicrobial resistance (AMR) is recognized as a major global health threat. In 2019, an estimated 1.27 million deaths were directly caused by antibiotic-resistant infections, with nearly 5 million associated deaths. Without urgent action, AMR-related mortality is projected to reach 10 million annually by 2050, with healthcare and economic losses exceeding US$1 trillion and US$100 trillion, respectively (Jesudason, 2024). In May 2024, WHO released an updated list of 24 priority drug-resistant pathogens classified as critical, high, or medium to guide treatment development and prevention strategies. Critical threats include carbapenem-resistant Gram-negative bacteria and drug-resistant *Mycobacterium tuberculosis*, whereas *Salmonella, Shigella*, *Pseudomonas aeruginosa*, *Staphylococcus aureus*, *Neisseria gonorrhoeae*, and *Enterococcus faecium* are listed as high-priority due to rising resistance and substantial public-health impact (World Health Organization, 2024). Ironically, the number of companies and laboratories working on antibiotic research has drastically declined, weakening the pipeline and endangering our capacity to control infectious illnesses. Future antibiotic availability will depend on innovation that goes beyond conventional discovery methods, backed by significant research and development (R&D) spending and a commercial environment that can encourage and maintain novel innovation (Cook and Wright, 2022).

Nanotechnology has rapidly transformed from a theoretical concept into one of the most powerful drivers of innovation across medicine, biotechnology, and materials science. Nanotechnology refers to the design and manipulation of materials at the 1–100 nm scale, where quantum and surface-area effects generate novel physical, chemical, and biological properties. Nanoparticles in drug delivery enhance solubility, stability, bioavailability, and targeted release by protecting drugs from degradation, prolonging circulation, and enabling tissue and cellular penetration, substantially improving therapeutic performance and reducing side-effects (Ochekpe et al., 2009). Nanotechnology is transforming medicine by enabling sharper diagnostics, improved imaging, and highly targeted drug delivery that reduces toxicity, especially in cancer therapy. Nanoparticles are now used in advanced pharmaceuticals, implants, and tissue-engineering materials. Wearable Nano-sensors can track vital signs and infection markers in real time, giving clinicians faster, more accurate insights. In general, nanotechnology strengthens modern diagnostics, therapeutics, regenerative medicine, and real-time patient monitoring (Haleem et al., 2023). However, many physico-chemical methods for nanoparticle synthesis involve hazardous reaction conditions and toxic or corrosive chemicals, which can negatively impact the environment. Therefore, green synthesis approaches have been developed as safer and more environmentally friendly alternatives for producing nanoparticles (Ghosh et al., 2021a). The biogenic synthesis of nanoparticles, using biological entities or extracts as reducing and stabilizing agents, offers a cost-effective and eco-friendly alternative to traditional chemical and physical methods, thereby being classified as a green synthesis strategy (Patil and Chandrasekaran, 2020).

In recent years, biogenic nanoparticles (BNPs) have become one of the most promising candidates in drug discovery, largely because they combine sustainable, biologically driven synthesis with strong antimicrobial potential. Their production through plants, microbes, and other biological systems results in nanoparticles that typically show better biocompatibility and lower toxicity compared to those produced by chemical routes (Shahzadi et al., 2025; Abdel-Megeed et al., 2025). Several recent studies demonstrate that BNPs can act through multiple antibacterial pathways including membrane disruption, oxidative stress induction, metabolic interference, and inhibition of biofilm development, making them particularly relevant for exploratory studies targeting multidrug-resistant organisms that evade classical antibiotics (Mondal et al., 2024; Arora et al., 2024). Because of these advantages, BNPs are now being explored across a wide range of applications, from antimicrobial therapeutics and targeted drug delivery to biosensing, environmental remediation, and agricultural protection (Parvin et al., 2025). Nevertheless, progress in the field will depend on improving the consistency of green-synthesis methods, gaining clearer mechanistic insight, and strengthening safety assessments to support their responsible use in biotechnology and medicine (Abdel-Megeed et al., 2025).

Nanoparticles are widely utilized in biological systems due to their distinctive physicochemical features. Their nanoscale dimensions provide an exceptionally high surface-area-to-volume ratio, which enhances their reactivity and facilitates efficient interaction with cellular components. These properties, combined with their structural stability and ability to be functionalized, underpin their growing use in diagnostic, therapeutic, and bioengineering applications (Nejatzadeh et al., 2021). Although nanotechnology offers a promising pathway for antimicrobial innovation, conventional chemically synthesized nanoparticles are frequently limited by possible hazardous effects. Some nanoparticles are known of their magnetic properties can cause toxic effects due to their small size, high reactivity, and ability to enter organisms and accumulate in tissues. Their environmental impact is rising, affecting aquatic life and respiratory systems. Toxicity depends on particle properties, ion release, and environmental factors like pH, salinity, and organic matter (Khan et al., 2019). However, biogenic nanoparticles, which are nanomaterials synthesized through biological systems or isolated biomolecules, generally exhibit markedly lower ecological and cytotoxic impacts than chemically synthesized nanoparticles and their green-synthesis paradigm also aligns with global priorities for eco-friendly, low-energy, and sustainable nanotechnology platforms (Shahzadi et al., 2025; Abdel-Megeed et al., 2025).

Despite the rapid expansion of research on BNPs, several critical knowledge gaps continue to hinder their scientific advances and their applications. First, green-synthesis methodologies remain highly variable, with differences in biological sources, extraction protocols, reaction conditions, and reducing agents leading to inconsistent particle size, morphology, and surface chemistry (Singh et al., 2023; Fahim et al., 2024). Such variability limits reproducibility and complicates comparative evaluation across studies. Second, regarding antimicrobial activity, there is an insufficient mechanistic comprehension of how BNPs engage with microbial cells at the molecular, genomic, proteomic, and metabolic levels. While many studies report antimicrobial activity, few provide mechanistic depth beyond general membrane disruption or ROS generation (Mondal et al., 2024; Arora et al., 2024). Third, toxicological and regulatory considerations remain underdeveloped, particularly regarding long-term cytotoxicity, biodistribution, ecotoxicity, and environmental persistence, which are essential for safety assessment and clinical translation (Abdel-Megeed et al., 2025). Finally, there is an urgent need for systematic comparative analyses that integrate synthesis parameters, mechanistic evidence, and functional performance to guide the rational design of next-generation BNPs and define clear future research priorities, particularly in the use of BNPs for combating the growing dilemma of antibiotic resistance.

The current narrative review aims to critically examine and synthesize the expanding evidence about the potential of biogenic nanoparticles (BNPs) to counter the growing threat of antibiotic resistance. It brings together recent advances in the green and biologically mediated synthesis of metal and metal-oxide nanoparticles from plants, microbes, algae, and other biological systems, emphasizing how these sustainable routes shape nanoparticle characteristics and antimicrobial performance. This review critically evaluates recent advances in BNPs as emerging tools to combat antibiotic resistance. It analyzes biologically mediated synthesis routes and how they govern nanoparticle physicochemical properties and antimicrobial performance. The review integrates current mechanistic evidence on BNP–bacteria interactions, and evaluates their biomedical and biotechnological relevance. Finally, key limitations related to reproducibility, safety, scalability, and regulatory translation are highlighted to define priorities for future BNP-based antimicrobial development.

## 2 Biogenic nanoparticles: key features and AMR relevance

### 2.1 Sources and core physicochemical features of biogenic nanoparticles

**FIGURE 1:**
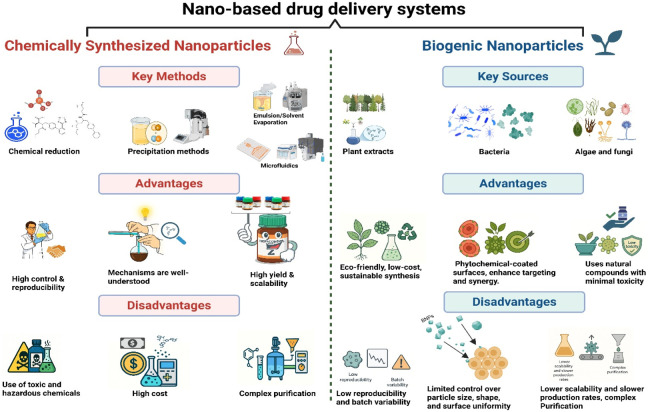
Advantages of BNPs over chemically synthesized nanoparticles. Legend: Biogenic nanoparticles (BNPs) are produced through biologically mediated synthesis routes using plants, microorganisms, or biomolecules, which act as reducing and stabilizing agents. Compared with chemically synthesized nanoparticles, BNPs are typically generated under mild conditions, lack toxic chemical reductants, and possess natural surface capping that enhances colloidal stability and biocompatibility. These features contribute to improved environmental safety and support their experimental use in antimicrobial and biomedical research (Created by the authors using BioRender.com).

BNPs are produced by living organisms, where natural metabolites act as reducing and capping agents to generate nanostructures with distinct physicochemical features (Iravani, 2011). These biologically driven processes often improve biocompatibility and provide intrinsic surface functionalization, offering a safer and more sustainable alternative to chemical synthesis (Ahmad et al., 2023). Chemically synthesized nanoparticles rely on strong reducing agents and harsh physicochemical conditions, often generating toxic residues and environmental hazards. In contrast, biogenic nanoparticles are produced using plants or microorganisms, where natural metabolites act as reducing and capping agents. This green route yields nanoparticles with higher biocompatibility, lower contamination, and improved environmental safety. Although biological methods may show some variability in size or yield, they remain more sustainable, cost-effective, and eco-friendly than conventional chemical synthesis (Figure 1) (Rahimi and Doostmohammadi, 2019).

Intrinsic biological metabolites, including phenolics, flavonoids, terpenoids, proteins, and polysaccharides, play a fundamental dual role in the biosynthesis of BNPs, acting both as reducing agents that convert metal ions into their nanoscale elemental forms and as capping agents that stabilize the resulting nanoparticles. Phenolics and flavonoids possess multiple hydroxyl groups capable of donating electrons, which efficiently reduce metal ions such as Ag^+^ or Au^3+^, while their aromatic structures strongly adsorb onto nanoparticle surfaces, preventing aggregation and conferring colloidal stability (Iravani, 2011; Adeyemi et al., 2022). Terpenoids similarly act as redox-active metabolites, contributing to metal-ion reduction and providing surface passivation that influences particle morphology and reactivity (El-Seedi et al., 2019). Proteins and peptides reduce metal ions through amino, carboxyl, and thiol groups, while simultaneously forming protective coronas that enhance biocompatibility and steric stabilization (Durán et al., 2015). Polysaccharides, including plant-derived and microbial exopolysaccharides, function as mild reducing agents due to their abundant hydroxyl groups and serve as highly effective capping materials through hydrogen bonding and chain entanglement, which prevent particle coalescence and support long-term stability (Tsekhmistrenko et al., 2020). Collectively, these metabolites create a biologically derived functional corona that enhances stability, modulates surface charge, and may even contribute additional antimicrobial synergy, making BNPs particularly well suited for applications targeting antimicrobial resistance.

### 2.2 Physicochemical features linked to antimicrobial function

At microbial scale, one of the defining attributes of BNPs is their small size and broad reactive surface, which enhance physicochemical interactions at the nano–bio interface. While the underlying synthesis mechanisms determine initial dimensions, the biological capping environment often gives rise to surface-enhanced reactivity that promotes rapid adherence to bacterial envelopes. This nanoscale proximity facilitates enhanced electron transfer, localized oxidative stress, and efficient access to periplasmic and cytoplasmic targets, effects that are difficult to achieve with bulk metals or chemically synthesized particles (Menichetti et al., 2023; Mendes et al., 2024). Importantly, BNP size is directly connected to their ability to generate reactive oxygen species (ROS) and penetrate biofilm matrices, phenomena strongly associated with antibacterial efficacy against multi-drug-resistant pathogens (MDR) (Babayevska et al., 2022).

Morphology adds an important dimension to the antimicrobial behavior of biogenic nanoparticles. Anisotropic shapes, such as triangular plates or rods, exhibit stronger antibacterial activity than spherical forms because their sharper edges and more reactive surface planes promote tighter contact with bacterial membranes and cause more severe structural disruption. These geometry-driven effects have been clearly demonstrated for triangular and rod-like silver nanoparticles, which deform bacterial envelopes more strongly than spherical particles (Pal et al., 2007; Van Dong et al., 2012). Unlike traditional antibiotics that rely on receptor compatibility, BNP–pathogen interactions occur through topology-driven mechanical and electrochemical forces, offering intrinsic advantages against bacteria that have remodeled their cell walls to evade antibiotics.

**FIGURE 2:**
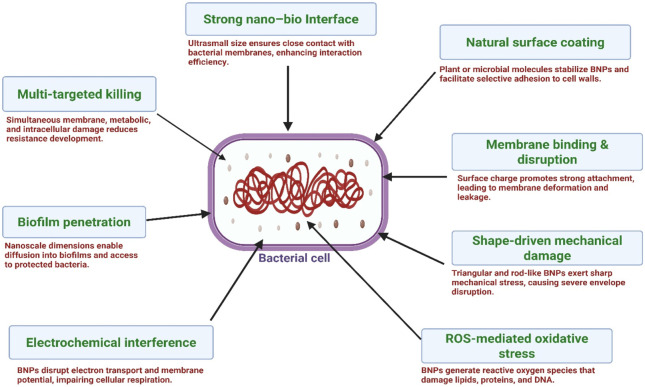
Mechanistic pathways of BNPs against bacterial cells. Legend: BNPs exert antibacterial effects through multiple parallel mechanisms, including membrane adhesion and permeabilization, reactive oxygen species generation, disruption of metabolic and enzymatic processes, damage to proteins and nucleic acids, and interference with quorum sensing and biofilm formation. Together, these actions underpin activity against Gram-positive and Gram-negative bacteria, including multidrug-resistant strains, largely demonstrated in vitro and limited preclinical studies (Created by the authors using BioRender.com).

A defining characteristic of biogenic nanoparticles is the natural organic layer that forms on their surface during green synthesis. This layer—composed of plant-derived metabolites or microbial biomolecules—acts as a functional coating that stabilizes the particle, enhances its surface charge, and determines its hydrophilic or hydrophobic behaviour. These surface attributes strongly influence how biogenic nanoparticles interact with bacterial cell envelopes. Mechanistic studies show that surface charge and interfacial chemistry govern adhesion to negatively charged lipopolysaccharide in Gram-negative bacteria and to teichoic-acid–rich regions of Gram-positive cell walls, ultimately shaping membrane disruption and antimicrobial efficiency (Singh et al., 2018). These interactions carry significant implications for AMR management, as cell-envelope remodeling is a hallmark of resistance in pathogens such as MRSA or carbapenem-resistant Enterobacteriaceae. BNPs exhibit enhanced colloidal stability due to their natural surface coronas composed of plant- or microbe-derived biomolecules, which act as capping and stabilizing agents. These biological coronas prevent nanoparticle agglomeration, preserve high surface energy, and maintain reactivity essential for antimicrobial action (Abuzeid et al., 2023). Additionally, the biological coatings improve biocompatibility and modulate interactions with microbial membranes, supporting prolonged antimicrobial performance in complex environments such as biofilms or chronic infections. Their naturally derived surface functionalities also facilitate safer biomedical applications compared with chemically synthesized nanoparticles (Abdel-Megeed et al., 2025). Crucially, BNPs exhibit superior biocompatibility relative to chemically synthesized nanoparticles because they lack toxic reducing agents and are naturally stabilized by plant- or microbe-derived surface compounds. Several *in vitro* and *in vivo* studies show that BNPs induce lower cytotoxicity, reduced inflammatory signaling, and diminished oxidative stress compared with nanoparticles synthesized using harsh chemical reducers such as sodium borohydride. For example, biogenic silver nanoparticles demonstrated significantly lower immunotoxicity and oxidative damage in mammalian cell models than their chemically derived counterparts (Składanowski et al., 2016). This improved safety profile is essential for translational AMR applications, including wound dressings, catheter coatings, and antimicrobial packaging. Figure 2 highlights the main antibacterial actions of BNPs.

### 2.3 Advances in biogenic synthesis and AMR-relevant properties

Recent advances in green synthesis have expanded the portfolio of biogenic metallic and metal-oxide nanoparticles, with plants, microbes, fungi, and algae now recognized as distinct biological “factories” capable of producing nanomaterials with tailored physicochemical traits. Earlier work on plant-mediated synthesis demonstrated that phytochemicals, particularly phenolics, flavonoids, terpenoids, and alkaloids, serve as effective reducing and stabilizing agents for silver, gold, copper oxide, and zinc oxide nanoparticles (Iravani, 2011; Singh et al., 2016). These metabolites strongly influence nucleation and growth, often yielding smaller, uniformly capped nanoparticles with enhanced stability and antimicrobial action. Comparative studies consistently show that plant-derived BNPs generate more reactive oxygen species (ROS) and exhibit superior membrane-disruptive activity than their chemically synthesized counterparts due to their biomolecule-rich coronas (Eker et al., 2025).

Microbial synthesis has similarly evolved, with bacteria and fungi enabling both intracellular and extracellular nanoparticle production. Fungal species, in particular, secrete high levels of reductase enzymes and proteins that facilitate controlled reduction of metal ions and form robust protein-capped nanoparticles with strong antibiofilm activity (Barapatre et al., 2016). Bacterial pathways, as demonstrated by species such as *Bacillus*, *Pseudomonas*, and *Lactobacillus*, often generate nanoparticles with distinct surface charges that enhance adhesion to Gram-positive or Gram-negative envelopes, thereby modulating antimicrobial potency (Singh et al., 2016). Comparative literature shows that microbe-derived BNPs typically exhibit slower but more controlled growth kinetics, which results in highly stable, densely capped nanoparticles with improved durability in biological environments.

BNPs synthesized from diverse biological systems have *shown robust antimicrobial activity against clinically relevant pathogens*. Fungal-derived silver nanoparticles, particularly those produced by Aspergillus flavus and Emericella nidulans, demonstrate strong bactericidal and antibiofilm properties, largely attributed to their protein-rich capping layers that enhance membrane disruption and oxidative stress (Barapatre et al., 2016). Recent evidence also highlights the efficacy of algae-mediated nanoparticles. Silver nanoparticles biosynthesized using Red Sea marine algae including *Ulva rigida*, *Cystoseira myrica*, and *Gracilaria foliifera* exhibited clear antimicrobial activity, with inhibition zones reaching up to 40 mm against Trichophyton mentagrophytes and notable effects against *Trichosporon cutaneum* and *Escherichia coli* (Algotiml et al., 2022). These findings confirm that algal metabolites, such as polysaccharides, phenolics, pigments, and proteins, function as reducing and stabilizing agents that generate highly bioactive nanoparticle surfaces capable of disrupting pathogenic microorganisms.

Algal metabolites provide a rich biochemical matrix that strongly influences the nucleation and growth of biogenic nanoparticles, producing materials with characteristic surface chemistries and improved dispersion stability. Soureshjani et al. (2021) demonstrated that algae-mediated synthesis of cuprous oxide nanoparticles yields particles with controlled morphology and enhanced colloidal stability due to algal-derived biomolecules acting as reducing and stabilizing agents. These algae-derived BNPs also exhibited clear antibacterial activity, indicating that algal metabolic components contribute directly to the functional bioactivity of the nanoparticles. Despite these advantages, algae-based synthesis remains comparatively less explored than plant- or microbe-mediated routes, representing an important but underdeveloped area in BNP research. Collectively, the literature demonstrates that advances in biogenic synthesis are not merely environmentally friendly alternatives to chemical reduction, they actively shape the structure, chemistry, and antimicrobial behavior of the resulting nanoparticles. A full understanding of these biogenic pathways is therefore critical to rationally designing BNPs with optimized antimicrobial performance.

## 3 Mechanisms of biogenic nanoparticle synthesis

Biogenic nanoparticle synthesis leverages on biological molecules to produce nanomaterial that are eco-friendly and efficient. This process involves distinct biochemical pathways and the activity of metabolites derived from plant and microorganisms.

### 3.1 Biochemical pathways of biogenic nanoparticle synthesis

**FIGURE 3:**
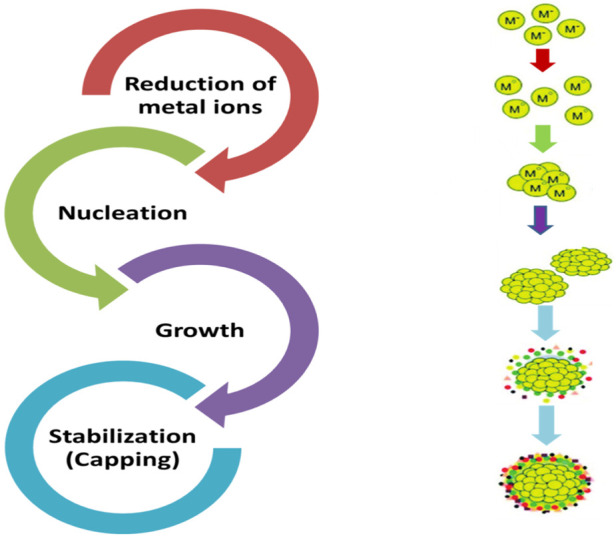
Schematic of green synthesis pathway showing reduction and stabilization. Legend: Schematic illustration of the biogenic (green) synthesis of nanoparticles, highlighting metal-ion reduction mediated by biological metabolites, followed by nucleation and growth of nanoparticles. Stabilization is achieved through capping by biomolecules such as phenolics, proteins, polysaccharides, or enzymes, which prevent aggregation and control particle size, morphology, and surface properties (Created by the authors using Microsoft PowerPoint).

Synthesis of BNPsinvolves the bio-reduction of metal ions which leads to the nucleation of tiny metal clusters to form nucleation centers. From these centers, the NPs then undergo growth via a process known as Ostwald ripening, leading to formation of larger particles. Finally, further aggregation is prevented by stabilizing agents present in the biological system which bind to the particle surface (Figure 3). In addition to prevention of further aggregation, the binding also results in maintenance of a stable, desired size and morphology (Cardoso et al., 2025a).

#### 3.1.1 Reduction of metal ions

This involves reduction of metal ions to their elemental state by biological entities (plant extracts and microorganism) using biomolecules as reducing agents. These reducing agents include plant derived phytochemicals (such as phenolics, polyphenols, terpenoids, alkaloids, and flavonoids) (Cardoso et al., 2025a), bioactive compounds derived from microorganism (Khan et al., 2019), proteins, exopolysaccharides and enzymes (Tsekhmistrenko et al., 2020; Lahiri et al., 2021; Jiang et al., 2025). Reduction can occur inside a cell (intracellular) or on its surface (extracellular), depending on the organism and conditions. These biomolecules donate electrons to metal ions, thereby converting them into neutral atoms (Abuzeid et al., 2023; Acharya et al., 2024).

Biosynthesis of nanoparticles (NPs) by bacteria is mediated by enzymes located on the cell membrane of bacteria which reduces metal ions to form NPs (Tsekhmistrenko et al., 2020) or by biomolecules produced by the bacteria (Cardoso et al., 2025b).

Microbial enzymes such as hydrogenase, nitrate reductase, oxidoreductases, laccase, reductase and peroxidase facilitate the transformation of metal ions into NPs by acting as electron donors (Rajeshkumar et al., 2021; Cardoso et al., 2025b). In addition to their role in reduction of metal ions, enzymes also play pivotal roles in nucleation, growth and stability of the synthesized NPs (Rajeshkumar et al., 2021).

#### 3.1.2 Nucleation

After reduction, the unstable metal atoms aggregate to form small clusters, thereby initiating nucleation. These small clusters serve as the initial nucleation centers for the nanoparticles. The rate and extent of nucleation are dependent on the concentration of reducing agents, type of reducing agents, pH and temperature (Adeyemi et al., 2022; Abuzeid et al., 2023).

#### 3.1.3 Growth

The nuclei formed grow into nanoparticles through subsequent addition of reduced metal atoms. The growth patterns involve the coalescence of smaller particles or through a process known as Ostwald ripening, where smaller particles dissolve and their materials are deposited onto the larger particles resulting in increased overall size and stability. The growth phase is modulated by the availability of reducing agents and stabilizers, which can control particle size and morphology (El-Seedi et al., 2019; Adeyemi et al., 2022; Khan et al., 2022).

#### 3.1.4 Stabilization (Capping)

The synthesized nanoparticles tend to aggregate due to their high surface energy. Biomolecules act as stabilizing agents or capping agents to prevent this by capping the surface of the nanoparticles, thereby providing stability. These biomolecules are typically the same biomolecules (phytochemicals, exopolysaccharides or proteins) that acted as reducing agents. This capping is crucial for maintaining colloidal stability and functional properties as forms a protective layer around the nanoparticle, providing electrostatic repulsion and steric hindrance (Guilger-Casagrande and Lima, 2019; Adeyemi et al., 2022; Abuzeid et al., 2023).

Biosynthesis of BNPs mediated by microorganism can also occur intracellular, in which natural metabolic processes such as photosynthesis, respiration, or nitrogen fixation are used for NPs synthesis (Khan et al., 2019). Intracellular synthesis of NPs in bacteria is mediated by the electrostatic interaction between metal ions attracted to the negatively charged bacterial membrane (Cardoso et al., 2025b).

In yeast, intracellular biosynthesis begins with passive diffusion of metal ions into the cell facilitated by the electrostatic interaction between the negatively charged yeast cell wall components and positively charged metal ions. Once inside the cell, the metal ions are reduced by membrane-bound oxidoreductases and quinones (Roychoudhury, 2020; Cardoso et al., 2025a).

### 3.2 Role of phenolics, proteins, and flavonoids in reduction and capping

Phytochemicals such as carotenoids, flavonoids, and phenolics have been used as capping to stabilize synthesized NPs (Yousefzadeh-Valendeh et al., 2023). Phenolics and flavonoids act as potent reducing agents owing to their multiple hydroxyl groups which facilitate electron transfer. Their role as capping agents is associated with their aromatic structures which allow strong binding to surfaces of nanoparticle (Ahmad et al., 2017; El-Seedi et al., 2019; Adeyemi et al., 2022; Abuzeid et al., 2023).

Proteins and peptides act as reducing and stabilizing agents. Their functional groups like thiols and amines allows them to reduce metal ions while binding through amino, carboxyl, or disulfide groups allows them to stabilize nanoparticles by preventing aggregation (Durán et al., 2015; Guilger-Casagrande and Lima, 2019; Adeyemi et al., 2022). Additionally, protein capping BNPscan also acts in the anchoring of drugs for subsequent transport into cells (Guilger-Casagrande and Lima, 2019).

Proteins, particularly enzymes play pivotal role as the foremost entities involved in BNPssynthesis. They are responsible for reduction and capping of metals in microorganism via redox reactions acting often as the nucleation sites (Ghosh et al., 2021b).

Aside enzymes, other proteins and peptides also play roles in capping and stabilization of the formed BNPs. Additionally, transport proteins are essential in intracellular entry of metal ions (Ghosh et al., 2021b). Furthermore, certain proteins such as Mms6 (a small acidic protein) have been used to control the size and shape of BNPs (Prozorov et al., 2007).

Extracellular polymeric substances secreted by bacteria such as siderophores, and metal-binding proteins play crucial role in stabilization of the NPs synthesized. Furthermore, these substances also influence the physicochemical properties of the NPs, prevent aggregation and enhance biocompatibility (Cardoso et al., 2025a).

### 3.3 Efficiency of green synthesis vs. chemical synthesis

Compared to chemical synthesis, green synthesis of nanoparticles has proven to be an efficient process as it is simple, cost effective, energy-efficient, eco-friendly and requires low energy input (Table 1) (Abuzeid et al., 2023; Cardoso et al., 2025a). Green-synthesized nanoparticles often exhibit higher surface area, superior stability, and enhanced biological activities compared to chemically synthesized nanoparticles (Aravind et al., 2021; Rahimzadeh et al., 2022).

**TABLE 1:** Key features of green/biogenic and chemical synthesis.

Feature	Green/Biogenic synthesis	Chemical synthesis	References
Reducing agents	Phenolics, flavonoids, proteins	Chemical reductants (e.g., NaBH4)	Abuzeid et al. (2023)
Capping/Stabilization agents	Biomolecules	Synthetic agents	Adeyemi et al. (2022)
Environmental impact	Low, eco-friendly	High, toxic by-products	Acharya et al. (2024)
Energy requirement	Low, ambient conditions	High, often elevated temperatures	Khan et al. (2022)
Product stability	High (natural capping)	Variable	Rahimzadeh et al. (2022)

A study conducted by Sreelekha et al. (2021) revealed that green-synthesized Ag NPs from *Mussaenda frondosa* leaf extract had a wider size range (30–60 nm) and were produced in a range of shapes (rod, spherical, triangle, and quasi-spherical). While the chemically synthesized NPs had smaller sizes range (9–14 nm) and were spherical in shape.

However, the green-synthesized ZnO NPs produced by Mohammadi and Ghasemi (2018) using *Musa acuminate* peel extract revealed a size range between 20 and 90 nm and were triangular and spherical in shape, while the chemically synthesized NPs had a wider size range between 20 and 200 nm, irregular morphology and a higher aggregation degree.

Green synthesized Ag NPs demonstrated greater stability compared to chemically synthesized NPs (Sreelekha et al., 2021). Capping of green NPs by positively charged organic molecules is responsible for their higher stability (Cardoso et al., 2025b).

## 4 Antibacterial mechanisms of biogenic nanoparticles

BNPs are primarily composed of metals such as Silver (Ag), Zinc Oxide (ZnO), Copper oxide (CuO), Titanium oxide (TiO_2_), Gold (Au), and Iron (III) Oxide (Fe_3_O_4_) (Moradi et al., 2023; Nikolova et al., 2023). They exhibit significant multifaceted antibacterial properties through several mechanisms with their efficiency highly influenced by particle size, shape and surface charge. Understanding these mechanisms is crucial in developing effective antimicrobial agents, especially in this era that is marked by increasing antibiotic resistance (Singh et al., 2018).

One of the primary mechanisms through which biogenic nanoparticles exert their antibacterial activity is by generating reactive oxygen species (ROS), which leads to oxidative stress in microbial cells. For instance, ZnO nanoparticles can produce ROS under both illuminated and dark conditions, exacerbating oxidative stress that damages cellular components including lipids, proteins, and DNA (Prasanna and Vijayaraghavan, 2015; Burmistrov et al., 2021). However, some studies suggest that the ROS generated by ZnO, plays a minor role, with metabolic disruption being more significant (Kadiyala et al., 2018; Godoy-Gallardo et al., 2021).

Similarly, CuO nanoparticles also exhibit enhanced oxidative potential, particularly in nanosheet forms, which demonstrates the importance of surface reactivity in ROS formation (Gilbertson et al., 2016). Other BNPs such as Silver (Ag), Gold (Au), Titanium Oxide (TiO_2_), Iron (III) Oxide (Fe_3_O_4_), and Selenium (Se) NPs can also generate ROS, resulting in lipid peroxidation, protein oxidation, and DNA damage, ultimately causing cell death (Fanoro and Oluwafemi, 2020; Filipović et al., 2021; Godoy-Gallardo et al., 2021; Zhang et al., 2021; Krishnamoorthy et al., 2022; Aguilar-Garay et al., 2024).

Another crucial aspect of their antimicrobial action is the disruption of cell membrane integrity. Upon exposure to BNPs, bacterial cell membranes are compromised due to direct interactions with particle surfaces that may lead to membrane lipid peroxidation, ultimately causing cell lysis (Jayaram et al., 2017). NPs interact with bacterial membranes, causing increased permeability, membrane depolarization, and leakage of cytoplasmic contents. This is observed for Ag, ZnO, CuO, Fe_3_O_4_, and Se NPs, often visualized by electron microscopy and confirmed by leakage assays (Ramalingam et al., 2016; Fanoro and Oluwafemi, 2020; Gabrielyan et al., 2020; Zhang et al., 2021; Krishnamoorthy et al., 2022). Surface charge modulation, such as chitosan-coating, can enhance membrane disruption and antibacterial potency (Ahmad et al., 2017; Krychowiak-Maśnicka et al., 2024).

NPs can bind to and denature bacterial proteins and enzymes, particularly those containing thiol or carboxyl groups. Ag and Au NPs disrupt ribosomal function and inactivate key enzymes, while ZnO and CuO NPs can inhibit β-lactamases and other metabolic enzymes (Fanoro and Oluwafemi, 2020; Krishnamoorthy et al., 2022; Aguilar-Garay et al., 2024).

Direct interaction with DNA/RNA or indirect damage via ROS leads to impaired replication and transcription. Ag, Au, ZnO, and Se NPs have been shown to cause DNA fragmentation and inhibit gene expression, contributing to bactericidal effects (Fanoro and Oluwafemi, 2020; Filipović et al., 2021; Zhang et al., 2021; Krishnamoorthy et al., 2022; Aguilar-Garay et al., 2024).

Nanoparticles can interfere with bacterial communication (quorum sensing), which orchestrates biofilm development, virulence factor production, and coordinated stress responses in numerous pathogens, including *Pseudomonas aeruginosa* and *Vibrio species* (Miller and Bassler, 2001; Defoirdt et al., 2012; Sánchez-López et al., 2020). ZnO and Ag NPs have demonstrated quorum sensing inhibition, which is particularly relevant for biofilm-associated infections (Kadiyala et al., 2018; Fanoro and Oluwafemi, 2020; Krishnamoorthy et al., 2022). ZnO nanoparticles synthesized via biogenic routes have demonstrated quorum-sensing inhibition (QSI) in *P. aeruginosa* and related virulence attenuation, alongside antisense downregulation of QS-regulated phenotypes such as pyocyanin production and biofilm formation. BNPs have shown consistent QS-interfering effects, ranging from inhibition of signal synthesis (autoinducers), interference with receptor signaling, to enzymatic degradation of signals when QS-inactivating moieties are present on NP surfaces or in coatings (Evans et al., 2018; Ivanova et al., 2020; Sánchez-López et al., 2020). The anti-virulence approach targets bacterial virulence mechanisms, rather than killing bacteria directly, to reduce disease severity and biofilm formation. This strategy, aligned with quorum sensing disruption research, utilizes biogenic nanoparticles to interfere with bacterial communication, attenuating pathogenicity and resilience without exerting bactericidal pressure that drives resistance. By disrupting quorum sensing systems, biogenic nanoparticles can effectively control infections while minimizing the risk of promoting antibiotic resistance (Defoirdt et al., 2012; Sánchez-López et al., 2020; Bouyahya et al., 2022).

The antimicrobial efficiency of biogenic nanoparticles is intricately linked to their physicochemical properties such as size, shape, and surface charge. These parameters dictate how nanoparticles interact with bacterial cells, influence their mechanisms of action, and ultimately determine their antibacterial potency.

Smaller nanoparticles consistently demonstrate superior antibacterial activity. This is attributed to their higher surface area-to-volume ratio, which enhances their interaction with bacterial membranes and facilitates greater production of reactive oxygen species (ROS) (Da Silva et al., 2019; Dong et al., 2019; Huang et al., 2019; Babayevska et al., 2022; Menichetti et al., 2023; Mendes et al., 2024). For example, silver nanoparticles (Ag NPs) with diameters of 10–12 nm exhibit lower minimum inhibitory concentrations (MICs) and more effective bactericidal action compared to larger particles (Dong et al., 2019; Menichetti et al., 2023; Ershov and Ershov, 2024). Similarly, Zinc oxide (ZnO) and Selenium nanoparticles (SeNPs) show increased antibacterial efficacies as particle size decreases, with 5 nm ZnO NPs and 81 nm SeNPs displaying optimal activity (Da Silva et al., 2019; Huang et al., 2019; Babayevska et al., 2022; Mendes et al., 2024). The enhanced efficacy of smaller particles is also observed in Copper oxide (CuO) and Iron (III) oxide (Fe_3_O_4_) nanoparticles, where reduced size correlates with increased membrane disruption and ROS generation (Susithra et al., 2023; Khatoon et al., 2024; Shoudho et al., 2024).

The shape of nanoparticles significantly affects their antibacterial performance. Spherical and sheet-like nanoparticles often perform better than other morphologies due to their larger surface areas and more efficient cellular uptake (Babayevska et al., 2022; Sikder et al., 2022; Mendes et al., 2024). For instance, spherical ZnO and AgNPs exhibit higher microbial cell reduction rates than flower- or rod-shaped counterparts, likely because their geometry that allows for more extensive contact with bacterial surfaces (Sikder et al., 2022; Babayevska et al., 2022; Menichetti et al., 2023 ; Mendes et al., 2024). Additionally, certain shapes, such as rods or sharp-edged structures, can physically penetrate bacterial membranes more effectively, thereby enhancing its antibacterial action (Babayevska et al., 2022; Sikder et al., 2022; Al-Otibi et al., 2023).

The surface charge of NPs typically measured as zeta potential, determines the electrostatic interactions between nanoparticles and bacterial cell walls. Nanoparticles with moderate surface charges (either slightly positive or negative) tend to exhibit optimal antibacterial activity (Huo et al., 2016; Rezazadeh et al., 2020; Khatoon et al., 2024; Krychowiak-Maśnicka et al., 2024; Shoudho et al., 2024; Wang Y. et al., 2025). Very high surface charge on nanoparticles can paradoxically reduce their antibacterial effectiveness due to excessive attraction, causing nanoparticles to aggregate rather than evenly interacting with bacterial cells, thereby diminishing their ability to target and disrupt bacterial functions (Krychowiak-Maśnicka et al., 2024). For example, AgNPs with zeta potentials around −21.5 to +14.9 mV showed the highest antibacterial activity, while those with extreme charges were less effective (Krychowiak-Maśnicka et al., 2024). Surface functionalization, such as with chitosan or polyphenols, can further modulate charge and enhance both stability and bioactivity (Rezazadeh et al., 2020; Marangon et al., 2021; Huo et al., 2016) (Table 2).

**TABLE 2:** Antibacterial mechanisms of major biogenic nanoparticles.

Nanoparticle	Antibacterial mechanisms	References
Silver (Ag)	Disruption of cell membrane integrity, generation of reactive oxygen species (ROS), DNA degradation, protein oxidation, ATP depletion, lipid peroxidation, release of Ag^+^ ions, interference with cellular enzymes and biomolecules	El-Shafai et al., 2019; Fanoro and Oluwafemi, 2020; Bruna et al., 2021; Godoy-Gallardo et al., 2021; Nguyen et al., 2021; Alavi and Moradi, 2022; Chandraker & Kumar, 2022; Vasiliev et al., 2023; Hao et al., 2024; Pernas-Pleite et al., 2025
Zinc oxide (ZnO)	ROS generation, Zn^2+^ ion release, membrane damage, electrostatic interaction with cell wall, metabolic pathway disruption, inhibition of biofilm formation	Agarwal et al., 2018; Kadiyala et al., 2018; El-Shafai et al., 2019; Godoy-Gallardo et al., 2021; Ifijen et al., 2022; Mendes et al., 2021; Summer et al., 2024
Copper Oxide (CuO)	ROS generation, Cu^2+^ ion release, membrane damage, protein and DNA interaction, enhanced effect when combined with Ag, internalization into cells	Godoy-Gallardo et al., 2021; Alavi and Moradi, 2022; Vasiliev et al., 2023; Hao et al., 2024; Summer et al., 2024; Yancey-Gray et al., 2025
Titanium dioxide (TiO_2_)	Photocatalytic ROS generation (especially under UV light), membrane damage, disruption of metabolic processes, DNA damage	Agarwal et al., 2018; Godoy-Gallardo et al., 2021; Mendes et al., 2021; Summer et al., 2024
Gold (Au)	Membrane interaction, ROS generation, protein and DNA binding, less potent than Ag but can enhance effects in two metallic forms	Fanoro and Oluwafemi, 2020; Godoy-Gallardo et al., 2021; Nguyen et al., 2021
Iron (III) oxide (Fe_3_O_4_)	ROS generation, disruption of redox balance, membrane potential alteration, ATPase activity modulation, concentration-dependent effects on bacterial growth	Gabrielyan et al., 2019; Godoy-Gallardo et al., 2021; Tiwari et al., 2024
Selenium (Se)	ROS generation, membrane damage, interference with thiol-containing enzymes, DNA and protein interaction	Ifijen et al. (2022)

## 5 Biogenic nanoparticles against MDR bacteria

MDR bacteria are defined as strains that no longer respond to at least one drug in three or more different classes of antibiotics. When bacteria acquire this level of resistance, treatment becomes challenging because many standard antimicrobial therapies are no longer effective (Magiorakos et al., 2012). Biogenic nanoparticles, especially Silver (Ag), Gold (Au), Copper (Cu), Zinc oxide (ZnO), and Selenium (Se), have demonstrated potent antibacterial activity against MDR ESKAPE pathogens (*Escherichia coli*, *Staphylococcus aureus*, *Klebsiella pneumoniae*, *Pseudomonas aeruginosa*, *Enterococcus faecium*, *Acinetobacter baumanii*) in both *in vitro* and some *in vivo* studies. These nanoparticles disrupt bacterial membranes, generate ROS, and often show synergy with antibiotics, reducing required drug concentrations and overcoming resistance.

*In vitro* studies consistently demonstrate that biogenic silver (Ag), gold (Au), copper (Cu), zinc oxide (ZnO), and selenium (Se) nanoparticles inhibit the growth and biofilm formation of MDR ESKAPE pathogens. In many cases, the reported minimum inhibitory concentrations (MICs) and minimum bactericidal concentrations (MBCs) are comparable to or lower than those of conventional antibiotics, highlighting their potential as experimental adjuncts or lead platforms for future antimicrobial development (Ashajyothi et al., 2016; Hossain et al., 2019; Scandorieiro et al., 2022; Da Cunha et al., 2023; Khan et al., 2023; Zeraatkar et al., 2022). *In vivo* evidence (e.g., murine wound models) supports the efficacy of certain biogenic nanoparticles such as Berberine/Chlorogenic acid NPs and Ag NPs, in promoting wound healing and reducing bacterial load, especially for Methicillin Resistant *S. aureus* (MRSA) (Fu et al., 2024; Hossain et al., 2019). Ag NPs: MICs for MDR ESKAPE pathogens typically range from 1 to 64 μg/mL, with MBCs often close to MICs, indicating strong bactericidal action (Khan et al., 2023; Liao et al., 2019; Da Cunha et al., 2023; Qais et al., 2019; Scandorieiro et al., 2022). Au NPs: show inhibition zones of 9–21 mm against ESKAPE isolates, with activity dependent on size and shape (Sultan and Saleh, 2025; Lee et al., 2019). Cu NPs/ZnO NPs: MICs range from 2 to 128 μg/mL, with Cu NPs often more potent than ZnO NPs, especially against biofilms (Ashajyothi et al., 2016). SeNPs: Effective at lower concentrations than antibiotics against MDR *P. aeruginosa* and *A. baumannii* (Zeraatkar et al., 2022) (Table 3).

**TABLE 3:** Examples of biogenic nanoparticles (BNPs) effective against MDR bacterial strains.

Nanoparticle	Bacteria	MIC(μg/mL)/Zone of inhibition	MBC(μg/mL)	References
AgNPs	*E. coli, S. aureus* (MRSA), *K. pneumoniae*, *P. aeruginosa, E. faecium*, *A. baumannii*	1–64 (MIC); 9–28 mm (zone)	2.8–64	Hossain et al., 2019; Da Cunha et al., 2023; Scandorieiro et al., 2022; Fu et al., 2024
AuNPs	*S. aureus, P. aeruginosa, A. baumannii, E. faecium, E. coli, K. pneumoniae*	9–21 mm (zone)	Not specified	Lee et al., 2019; Sultan and Saleh, 2025
CuNPs	*S. aureus, K. pneumoniae, P. aeruginosa*	2–128 (MIC)	Not specified	Ashajyothi et al. (2016)
ZnONPs	*S. aureus, K. pneumoniae, P. aeruginosa*	2–128 (MIC)	Not specified	Ashajyothi et al. (2016)
SeNPs	*P. aeruginosa, A. baumannii*	Not specified	Not specified	Zeraatkar et al. (2022)
Berberine/Chlorogenic acid NPs	*S. aureus* (MRSA)	1.5 (MIC, µM)	Not specified	Fu et al. (2024)

Synergistically, many studies report that combining NPs with antibiotics reduces MICs up to 32-fold and enhances biofilm eradication (Mishra et al., 2024; Tawre et al., 2022; Yassin et al., 2022; Tahan et al., 2023; Fu et al., 2024).

## 6 Synergistic effects and nanocomposite systems

Nanocomposite systems combining BNPswith antibiotics, essential oils, or phytochemicals offer enhanced antibacterial activity and modulation of resistance through different molecular mechanisms (Table 4). These strategies have proven to be promising in combating MDR pathogens and reducing antibiotic reliance. The synergistic effects arise from multiple mechanisms such as: disruption of bacterial membrane thereby facilitating antibiotic and phytochemical uptake (Fahmy et al., 2025; Pernas-Pleite et al., 2025), disruption of biofilms, allowing the co-agents to access and kill embedded bacteria (Aabed and Mohammed, 2021; Fahmy et al., 2025), generation of reactive oxygen species (ROS), which damage bacterial components and potentiate the action of co-agents (Francis et al., 2023; Marfavi et al., 2024; Pernas-Pleite et al., 2025), efflux pump inhibition through gene down-regulating (Fahmy et al., 2025) and controlling release and targeted delivery of essential oils or phytochemicals, hence improving their stability and bioavailability (Rai et al., 2017; Motelică et al., 2023; Murugan et al., 2025).

**TABLE 4:** Synergistic Combinations of BNPs with Antibiotics, Essential oils or Phytochemicals.

NP type	Co-agent	Target microbe	Observed synergy	References
AgNPs	Chloramphenicol, rifampicin and tetracycline	*E. coli*	Enhanced growth inhibition	Pernas-Pleite et al. (2025)
AgNPs	Ampicillin, penicillin G, colistin, kanamycin and streptomycin	*S. aureus*	Enhanced growth inhibition	Pernas-Pleite et al. (2025)
AgNPs	Ciprofloxacin	*S. aureus, E. coli, B. cereus, V. cholerae,* and *Proteus vulgaris*	Increased antibacterial activity	Bhat et al. (2015)
AgNPs and SeNPs	Piperacillin, piperzobactam, ceftazidime, colistin, aztreonam, meropenem, gentamicin and ciprofloxacin	MDR *P. aeruginosa*	Increased zones of inhibition	Fahmy et al. (2025)
AgNPs and SeNPs	Erythromycin, nitrofurantoin, tetracycline, clindamycin, chloramphenicol, quinapristin, linzolid, trimethoprim, ciprofloxacin, rifampicin and cefoxitin	Methicillin resistant *S. aureus*	Increased zones of inhibition	Fahmy et al. (2025)
AgNPs	Tetracycline	*Bacillus cereus, Bacillus subtilis, Enterobacter aerogenes, E. coli,* and *S. aureus*	Enhanced antibacterial activity	Fátima et al. (2016)
AgNPs	Ampicillin, kanamycin erythromycin and chloramphenicol	AgNPs biosynthesized using *Trichoderma viride E. coli, Salmonella* typhi*, S. aureus* and *Micrococcus luteus*	Enhanced antibacterial activity	Fayaz et al. (2010)
AgNPs	Bacitracin, ciprofloxacin, tetracycline, and cefixime	*E. coli*	Increased zones of inhibition	Aabed and Mohammed (2021)
AgNPs	Ciprofloxacin, tetracycline, and cefixime	*S. aureus*	Increased zones of inhibition	Aabed and Mohammed (2021)
AgNPs	Bacitracin and ciprofloxacin	*P. aeruginosa*	Increased zones of inhibition	Aabed and Mohammed (2021)
AgNPs	Azithromycin, gentamicin, oxacillin, cefotaxime, neomycin, ampicillin/sulbactam, cefuroxime, fosfomycin, chloramphenicol and oxytetracycline	*Salmonella* species	Increased antibacterial activity	Abo-Shama et al. (2020)
AgNPs	Azithromycin, cefotaxime, cefuroxime, fosfomycin and chloramphenicol	*E. coli* and *S. aureus*	Increased antibacterial activity	Abo-Shama et al. (2020)
ZnONPs	Azithromycin, cefotaxime and oxytetracycline	*E. coli S. aureus* and *Salmonella* species	Increased antibacterial activity	Abo-Shama et al. (2020)
AgNPs	Neomycin and tetracycline	Drug resistant *Salmonella* typhimurium	Enhanced antibacterial activity	McShan et al. (2015)
AgNPs	Cinnamon essential oil	Biofilm forming MDR isolates of *Streptococcus agalactiae*	Decrease in MIC values	Abd El-Aziz et al. (2021)
AgNPs	Oregano essential oil	Enteroaggregative strain of *E. coli* and KPC producing strain of *K. pneumoniae*	Disruption of preformed biofilms and prevention of biofilm formation	Scandorieiro et al. (2023)
AuNPs	Lavender essential oil	*P. mirabilis*	Eradication of biofilm	Fadel et al. (2023)
MgONPs	Clove and thyme essential oils	*S. aureus, E. faecalis,* and *E. coli*	Enhanced antibacterial activity	Manaa et al. (2025)
AgNPs	*Kelussia odoratissima* essential oil	*Listeria monocytogenes, Salmonella enterica* and *P. aeruginosa*	Enhanced antibacterial activity	Oroojalian et al. (2017)
AgNPs	*Teucrium polium* essential oil	*E. coli O157:H7, Salmonella enterica* and *P. aeruginosa*	Enhanced antibacterial activity	Oroojalian et al. (2017)
AgNPs	*Acanthospermum austral* essential oil	*M. canis, N. gypsea and M. globosa*	Enhanced antimicrobial activity	Mussin and Giusiano (2024)
AgNPs	Eucalyptus essential oil	*S. enterica, E. coli,* and *B. subtilis*	Bacterial growth inhibition	Heydari et al. (2017)
AuNPs	*Nigella sativa* essential oil	*S. aureus*	Inhibition of growth and biofilm formation	Manju et al. (2016)
AgNPs and ZnONPs	Rosemary and oregano essential oils	*L. monocytogenes, S. aureus, E. coli,* and *S.* Typhimurium	Enhanced antibacterial efficacy	Morsy et al. (2014)
AgNPs	Ursolic acid	*E. coli*, *P. aeruginosa*, *S. aureus* and *Streptococcus epidermidis*	Reduction in bacterial growth	Wrońska et al. (2023)
AgNPs	Carvacrol and thymol	Enteroaggregative strain of *E. coli* and KPC producing strain of *K. pneumoniae*	Disruption of preformed biofilm and prevention of biofilm formation	Scandorieiro et al. (2023)
AgNPs	*Vatica diospyroides* cotyledon extract	*S. aureus* and *Bacillus subtilis*	Enhanced antibacterial activity	Musimun et al. (2022)

Synergistic activity of AgNPs synthesized using *Candida albicans* and antibiotic was reported by Bhat et al. (2015). Increased antibacterial activity against *S. aureus, E. coli, B. cereus, Vibrio cholerae,* and *Proteus vulgaris* was observed when ciprofloxacin was combined with the synthesized AgNPs. A study conducted by Pernas-Pleite et al. (2025) revealed synergistic activity of combining AgNPs produced using *Lysinibacillus* sp. broth with chloramphenicol, rifampicin or tetracycline against *E. coli*. Similar, they also reported synergistic activity against *S. aureus* using combination of the AgNPs with ampicillin, penicillin G, colistin, kanamycin or streptomycin.

When combine with AgNPs or SeNPs, the antibacterial activities of piperacillin, piperzobactam, ceftazidime, colistin, aztreonam, meropenem, gentamicin and ciprofloxacin against MDR *P. aeruginosa* were increased. The percentage fold increase in zones of inhibition ranged from 45% to 182% for AgNPs combined with antibiotics and 41.7%–175% for SeNPs combined with antibiotics. Increased zones of inhibition were also observed against Methicillin Resistant *S. aureus* using combinations of the NPs (AgNPs or SeNPs) with erythromycin, nitrofurantoin, tetracycline, clindamycin, chloramphenicol, quinapristin, linzolid, trimethoprim, ciprofloxacin, rifampicin and cefoxitin. The increase in zones of inhibition was in the range of 28.3%–147% for AgNPs - antibiotics combinations and 17.3%–113.9% for SeNPs - antibiotics combinations (Fahmy et al., 2025).

Enhanced antibacterial activity of AgNPs when combined with tetracycline was reported by Fátima et al. (2016) against *B. cereus, Bacillus subtilis, Enterobacter aerogenes, E. coli,* and *S. aureus.* AgNPs biosynthesized using *Trichoderma viride* was observed to enhance the antibacterial activity of ampicillin, kanamycin erythromycin and chloramphenicol against *E. coli, Salmonella* Typhi, *S. aureus and Micrococcus luteus* (Fayaz et al., 2010). Increased zones of inhibition were observed when ampicillin, kanamycin, erythromycin, or chloramphenicol were used in combination with AgNPs with percentage fold increase in the range of 70.00%–81.82%, 10.00%–46.15%, 11.11%–29.17% and 10.00%–27.27% respectively.

Aabed and Mohammed (2021) reported substantial synergistic effects of combining AgNPs prepared from extracts of *Anastatica hierochuntica* (An-AgNPs) and *Artemisia absinthium* (Ar- AgNPs) with antibiotics. Increased zones of inhibition against *E. coli* were observed when bacitracin, ciprofloxacin, tetracycline, or cefixime were combined with An-AgNPs. Similarly, the activity of ciprofloxacin, tetracycline, and cefixime against *S. aureus* were enhanced when combined with An-AgNPs. Furthermore, combination of An-AgNPs with bacitracin or ciprofloxacin showed synergistic effect against *P. aeruginosa*. Combination of Ar-AgNPs with antibiotics also resulted in synergistic effects against *E. coli* and *P. aeruginosa*.

A study conducted by Abo-Shama et al. (2020) also revealed synergistic effects of combining antibiotics with BNPs (AgNPs and ZnONPs) against isolates of *E. coli, S. aureus*, and *Salmonella* species. Combination of AgNPs with azithromycin, gentamicin, oxacillin, cefotaxime, neomycin, ampicillin/sulbactam, cefuroxime, fosfomycin, chloramphenicol and oxytetracycline had synergistic effect against *Salmonella* species. Significant synergistic effects were also observed against *E. coli* and *S. aureus* when AgNPs was combined with azithromycin, cefotaxime, cefuroxime, fosfomycin and chloramphenicol. Similarly, significant increase in antibacterial activity was observed combining ZnONPs with antibiotics against *E. coli, S. aureus* and *Salmonella* species (Abo-Shama et al., 2020). Synergistic effects of combining AgNPs with neomycin or tetracycline against drug resistant *Salmonella* Typhimurium was reported also by McShan et al. (2015).

Synergistic interaction between essential oil of cinnamon and AgNPs against biofilm forming MDR isolates of *Streptococcus agalactiae* was reported by Abd El-Aziz et al. (2021). A significant decrease in MIC values was observed using cinnamon essential oil combined with AgNPs compared when they were used separately indicating synergistic inhibition of *S. agalactiae* isolates.

Combination of oregano essential oil with biogenic AgNPs was reported to exhibit activity against preformed biofilms and also prevents biofilm formation by Enteroaggregative strain of *E. coli* and KPC producing strain of *K. pneumoniae* (Scandorieiro et al., 2023). A nanosystem composed of lavender essential oil and AuNPs exhibited ability to penetrate and eradicate biofilm produced by *P. mirabilis*. The nano-system also demonstrated wound healing potential (Fadel et al., 2023).

Combination of phytosynthesized MgONPs with essential oils of clove and thyme demonstrated synergistic effects against *S. aureus, E. faecalis,* and *E. coli* in a study conducted by Manaa et al. (2025). Synergistic antimicrobial activities of combining AgNPs with essential oil of *Kelussia odoratissima* was reported against *Listeria monocytogenes, Salmonella enterica* and *P. aeruginosa* while essential oil of *Teucrium polium* acted synergistically with AgNPs against *E. coli O157:H7, S. enterica* and *P. aeruginosa* (Oroojalian et al., 2017). Similarly combination of AgNPs with essential oil of *Acanthospermum australe* exhibited synergistic activity against *Microsporum canis, Nannizzia gypsea and Malassezia globosa* (Mussin and Giusiano, 2024).

So also, AgNPs combined with essential oil of eucalyptus exhibited a synergistic effect by inhibiting the growth of *S. enterica, E. coli,* and *B. subtilis* (Heydari et al., 2017). Growth and biofilm formation by *S. aureus* were inhibited by AuNPs - *Nigella sativa* essential oil combination (Manju et al., 2016; Morsy et al., 2014) observed enhanced antibacterial efficacy against *L. monocytogenes, S. aureus, E. coli,* and *S.* Typhimurium when combinations of essential oils (rosemary and oregano EOs) and NPs (AgNPs and ZnONPs) were incorporated into pullulan films.

BNPshave also been reported to act synergistically with phytochemicals. Wrońska et al. (2023) reported synergistic activity of combining AgNPs with ursolic acid. The AgNPs-ursolic acid combinations significantly reduced the growth of *E. coli*, *P. aeruginosa*, *S. aureus* and *Streptococcus epidermidis*. Similarly, combinations of biogenic AgNPs with carvacrol or thymol (both of which are monoterpenoid phenols) showed ability to act against preformed biofilm of the test strains as well as prevent biofilm formation by the strains (Scandorieiro et al., 2023). Musimun et al. (2022) reported that a combination of *Vatica diospyroides* cotyledon extract with AgNPs exhibited strong synergistic activity against isolates of *S. aureus* and *Bacillus subtilis*.

## 7 Biotechnological and biomedical applications

**FIGURE 4:**
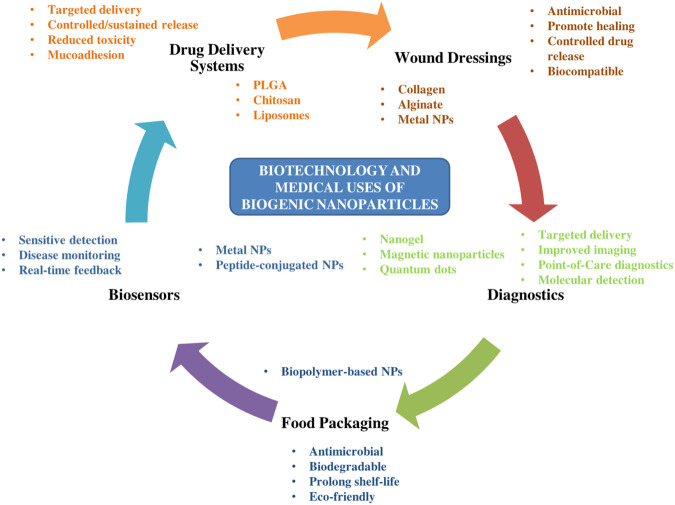
Application map showing biotechnology and medical uses of BNPs. Legend: Overview of the main biotechnology and biomedical application areas in which biogenic nanoparticles are being explored, including antimicrobial coatings, wound-related systems, drug-delivery research, biosensing and diagnostics, anticancer studies, and food-packaging materials. The applications shown represent active research and preclinical development rather than established clinical or industrial use (Created by the authors using Microsoft PowerPoint).

BNPsoffer multifunctional and sustainable solutions in wound dressing, drug delivery systems, biosensing, diagnostics and food packaging, with promising potential for targeted, biocompatible, and controlled-release applications (Figure 4). These properties in addition to their eco-friendly nature align with circular economy principles.

BNPsare considered bioavailable and biocompatible due to the presence of biologically derived capping agents and absence of toxic reducing agents, hence they are promising candidates in safer drug delivery, cancer therapy, and tissue engineering (Sreelekha et al., 2021; Cardoso et al., 2025a).

BNPs are also promising candidates as drug delivery systems, antimicrobials agents, anticancer drugs, and diagnostic agents due to their small size (which is associated with increased surface/volume ratio) and biocompatibility (Ghosh et al., 2021b). BNPs drug delivery systems can increase the solubility of drugs that are poorly water-soluble and prevent degradation of therapeutic agents thereby enhancing drug bioavailability (Ly et al., 2024).

Owing to their antimicrobial activity against multidrug-resistant pathogens and biocompatibility, BNPs are being explored as promising candidates in biomedical research, although translation to clinical use remains limited by safety, scalability, and regulatory challenges (Akshaykranth et al., 2021). Several BNPsof microbial origin such as NPs of Ag, Ag, Se, and Te have proven to be effective (*in vitro*) as antimicrobial and antibiofilm agents (El-Sheekh et al., 2020; Gursoy, 2020).

Low toxicity, high biocompatibility with natural polymers (collagen, chitosan, alginate), and biodegradability are qualities that makes BNPssuitable for potential medical and food applications (Mohammed et al., 2017; Si et al., 2020; Yudaev et al., 2022; Froelich et al., 2023; Kusnadi et al., 2024; Gunasena et al., 2025). For high specificity, BNPscan be functionalized with ligands (such as peptides and antibodies), enabling targeted delivery to disease sites while minimizing off-target effects (Chen et al., 2021; Zeb et al., 2022; Ilieş et al., 2024; Anwar et al., 2025).

Biosynthesized TiO_2_ NPs can be applied as double-edge material in biomedicine and purification of the environment because of their enhanced antibacterial activity and photocatalytic efficiency (Aravind et al., 2021). Biogenic TiO_2_NPs exhibited antimicrobial activity against bacterial and fungal pathogens, proving their potential as broad-spectrum nanomedicine (Pandya and Ghosh, 2024).

BNPs have emerged as effective anticancer agents and drug delivery systems, this is due to their excellent penetration ability which allows them to penetrate across membranes and barriers (such as blood–tissue and blood–brain barriers) (Gonzalez-Ballesteros et al., 2017). Furthermore, their ability to activate innate immunity, suppress tumor development and enhance antigen specific antitumor responses makes them promising candidates in cancer therapy (Dowaidar, 2023; Shah et al., 2025).

Polymeric nanoparticles (e.g., PLGA, chitosan, alginate) and stimuli-responsive systems enable sustained and on-demand drug release, thereby improving therapeutic efficacy and reducing side effects (Zeb et al., 2022; Mohammed et al., 2017; Zhou et al., 2021; Froelich et al., 2023; Anwar et al., 2025). Chitosan-stabilised AgNPs demonstrated enhanced antimicrobial activity and biocompatibility, proving their suitability for biomedical applications (Jiang et al., 2025).

Biogenic nanoparticles are also applicable in the development of biosensors for the detection of analytes, such as biomolecules, toxins and pathogens (Zimina et al., 2022; Shah et al., 2025). AuNP biosensors are used to detect ochratoxin A- a mycotoxin that commonly contaminate raw materials and food products (Popescu and Ungureanu, 2023). Biogenic AgNP-based sensor demonstrated high sensitivity and specificity in the detection of paracetamol on solution (Zamarchi and Vieira, 2021).

The unique optical properties, mechanical resistance, diffusivity, and solubility of BNPscontribute to their application in food industries as food packaging materials (Kumar et al., 2023). BNPsfood packaging materials improve food stability, prevent oxidation, and possess antimicrobial and UV protective properties (Hamad et al., 2018). The use of BNPs as packaging materials makes it possible to improve the antibacterial and mechanical properties of food packages as well as reduce their permeability to water vapor (Nwabor et al., 2020). A nanocomposite developed by Youssef et al. (2015) using chitosan/polyvinyl alcohol/TiO_2_NP demonstrated ability to prolong the shelf life of soft white cheese, suggesting their possible use as excellent food packaging material.

BNPs are produced using biological entities (plant or microorganism), this supports sustainable biotechnology as it reduces hazardous chemicals and energy use (Tolisano and Del Buono, 2023; Gunasena et al., 2025; Si et al., 2020; Nyandoro et al., 2024; Jain et al., 2024; Bhandari et al., 2023). BNPssynthesis aligns with circular economy as it promotes resource efficiency, waste reduction, and eco-friendly product life cycles (Tolisano and Del Buono, 2023; Bhandari et al., 2023; Jain et al., 2024).

## 8 Challenges and limitations

Despite the significant promise of biogenic nanoparticles, several limitations continue to restrict their scientific and translational advancement. A major challenge is the lack of standardized synthesis protocols: variations in biological sources, extraction methods, pH, temperature, and reaction kinetics frequently lead to inconsistent particle sizes, morphologies, capping profiles, and antimicrobial performance, making cross-study comparison and reproducibility difficult (Singh et al., 2023; Radulescu et al., 2023; Fahim et al., 2024; Shahzadi et al., 2025). This heterogeneity directly complicates regulatory evaluation and industrial scale-up. Furthermore, although many studies emphasize the superior biocompatibility of green/biogenic nanomaterials compared with chemically synthesized counterparts, long-term toxicological data remain incomplete. Critical aspects such as chronic cytotoxicity, immunological responses, biodistribution, bioaccumulation, and environmental persistence are still insufficiently characterized, and several reviews explicitly call for more rigorous *in vivo* and long-term safety assessments (Khan et al., 2019; Radulescu et al., 2023). Scalability also represents a substantial bottleneck: most green-synthesis processes have been optimized at laboratory scale and often lose yield, colloidal stability, and physicochemical uniformity when production is expanded, highlighting the need for robust process control and quality-by-design approaches (Fahim et al., 2024; Shahzadi et al., 2025). In parallel, regulatory frameworks for nanomaterials, particularly for biogenic and “green” nanoparticles, remain fragmented, with no globally harmonized standards for characterization, safety testing, and environmental risk assessment, which slows their path toward clinical and industrial deployment (Parvin et al., 2025).

Reproducibility and scalability are critical limitations in the development of biogenic nanoparticles. Biological synthesis relies on complex and variable inputs such as plant genotype, microbial strain, extraction procedure, and reaction parameters (pH, temperature, precursor concentration), leading to significant variability in size, morphology, surface chemistry, and bioactivity across batches. This variability hinders reproducibility within and between laboratories, complicates comparative analysis, and challenges robust standardization of biological nanoparticle production (Jiang et al., 2025. Scalability of green synthesis remains a major limitation. Protocols effective at laboratory scale often show reduced yield, altered physicochemical properties, and inconsistent stability upon scale-up. Batch-to-batch variability in biological extracts and nanoparticle characteristics further hinders reproducible manufacturing, compounded by the lack of robust process control, in-line quality monitoring, and optimized large-volume production systems. As a result, successful industrial-scale translation of green-synthesized nanomaterials remains limited and requires substantial process innovation (Gacem and Abd-Elsalam, 2022). Regulatory translation of biogenic nanoparticles remains poorly defined. Existing regulatory frameworks are fragmented and largely tailored to chemically synthesized nanomaterials, offering limited guidance for biologically derived particles. Major gaps include harmonized standards for physicochemical characterization, long-term toxicology, biodistribution, environmental risk assessment, and safety evaluation. In the absence of clear regulatory pathways and standardized reporting criteria, the safe and scalable translation of biogenic nanoparticles into healthcare applications remains challenging (Chaturvedi and Ranjan, 2025).

On the other side, the current narrative review is constrained by its reliance on previously published studies, many of which differ in synthesis conditions, characterization methods, biological models, and antimicrobial test protocols, limiting direct comparability across findings. The review does not generate new experimental or clinical data; therefore, key aspects such as long-term toxicity, biodistribution, and environmental fate of biogenic nanoparticles remain dependent on gaps and uncertainties in the existing literature rather than being experimentally resolved here. In addition, no quantitative meta-analysis was performed, so the strength of evidence for specific nanoparticle types, synthesis routes, or antimicrobial outcomes cannot be expressed in pooled effect sizes. Finally, issues related to regulatory translation, large-scale manufacturing, and techno-economic feasibility are only briefly discussed and warrant more detailed, multidisciplinary evaluation in future work.

## 9 Patented and commercial developments related to BNPs

**FIGURE 5:**
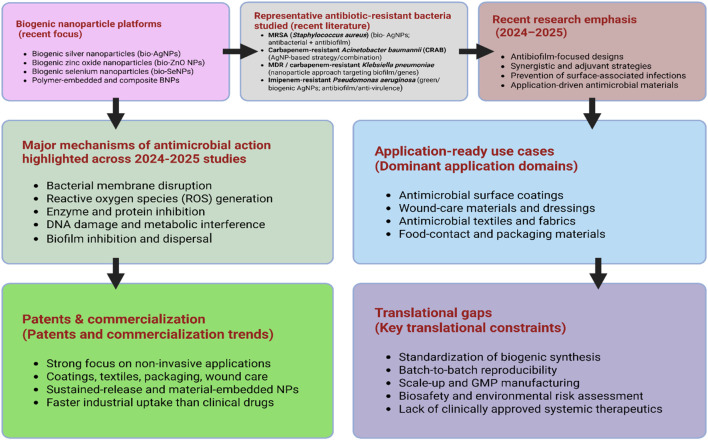
Schematic of the translational landscape of BNPs (2025 translational update) for antimicrobial applications, highlighting patents, application routes, and key gaps. Legend: The schematic depicts the translational landscape of biogenic nanoparticle–based antimicrobial strategies, integrating green synthesis platforms, key mechanisms of action, application-ready use cases, and patent/commercial trends. It highlights non-invasive applications where translation is most advanced, alongside major constraints such as standardization, scalability, biosafety, regulatory challenges, and the absence of clinically approved systemic therapies (Created by the authors using BioRender.com).

The translation of bio-inspired and biogenic nanoparticle platforms into patented technologies and commercial products has been enabled by nanoscale physicochemical advantages (e.g., high surface area and functional versatility) that support biomedical and industrial performance, with early successes exemplified by clinically deployed nanocarriers such as liposomes (Di paolo, 2004). In parallel, metallic nanoparticles (especially silver and gold) have driven substantial patenting and product-oriented innovation, including imaging/diagnostics (Sokolov et al., 2003) and antimicrobial materials, with silver nanoparticles showing antifungal activity against *Candida albicans* (Kim et al., 2009). Although patent analyses from 2007 to 2017 demonstrate that nanoparticle-based antimicrobial technologies are predominantly translated into antimicrobial textiles, fabrics, and related consumer products rather than systemic therapeutic applications (Sim et al., 2018). Table 5 summarizes recent patents and commercially oriented developments involving BNPs for controlling antibiotic resistant pathogens, highlighting target species, nanoparticle types, application domains, and translational status. In the last 5 years, BNPs synthesis has become more sharply defined, with recent reviews highlighting a shift toward antimicrobial coatings, wound-care materials, and food-safety or packaging applications that align with sustainability goals, while still encountering significant translational limitations (Jangid et al., 2025). Despite advances in mechanism-guided design and the development of tunable polymer–AgNP nanocomposite patents, antimicrobial coating technologies remain limited by unresolved challenges related to standardization, reproducibility, and scalable manufacturing. These limitations are further compounded in biologically mediated synthesis approaches by inherent variability in biological feedstocks and associated biomolecular components (Dube and Okuthe, 2025). However, advancing biogenic nanoparticle (BNP) patents and applications requires a shift toward mechanism-driven design informed by structure–activity relationships, together with harmonized synthesis and characterization protocols to enhance reproducibility. Controlling biological feedstocks, molecularly profiling bioreductants and capping agents, and applying quality-by-design principles are essential to reduce intrinsic variability. Progress will further depend on interdisciplinary collaboration to enable scalable manufacturing, robust safety assessment, and translational implementation (Ayub et al., 2025). As a result, the scarcity of clinically approved antimicrobial therapeutics based explicitly on fully BNPs highlights a continuing disconnect between experimental research, patent activity, and regulatory-approved medical products. Figure 5, provides a schematic overview of recent (2024–2025) advances in biogenic nanoparticle–based antimicrobial strategies, illustrating the relationship between nanoparticle platforms, bacterial targets, dominant application routes, patent activity, and key translational gaps.

**TABLE 5:** Recent patents and commercially oriented developments of biogenic nanoparticles for antimicrobial applications*.

BNP type	Biological source	Primary antimicrobial targets	Evidence level	Translational status	Key limitations	References
AgNPs	Plant extracts	MRSA, *E. coli, P. aeruginosa, A. baumannii*	*In vitro*; limited *in vivo* (wound models)	Preclinical; materials/devices	Batch variability, scale-up, long-term toxicity	Behravan et al., 2019; Scandorieiro et al., 2022
AgNPs	Bacteria/Fungi	MDR ESKAPE pathogens	*In vitro*; limited *in vivo*	Preclinical	Reproducibility, regulatory uncertainty	Barapatre et al., 2016; Pernas-Pleite et al., 2025
ZnONPs	Plant extracts	*S. aureus, K. pneumoniae, P. aeruginosa*	*In vitro*	Experimental	ROS-linked toxicity, stability	Zeghoud et al., 2022; Sirelkhatim et al., 2015
CuONPs	Plant/microbial	Gram-positive and gram-negative MDR bacteria	*In vitro*	Experimental	Cytotoxicity, environmental fate	Gudkov et al., 2024; Ren et al., 2009
SeNPs	Microbial/plant	*P. aeruginosa, A. baumannii*	*In vitro*; limited *in vivo*	Preclinical	Narrow safety window, limited PK data	Zeraatkar et al., 2022; Vinković Vrček, 2018
BNP-polymer composites	Plant/microbial BNPs + polymers	Surface-associated pathogens	*In vitro*; device-level testing	Translational (non-systemic)	Standardization, durability	Morsy et al., 2014; Omerović et al., 2021
BNP-enabled coatings	Various biogenic routes	Hospital and food-contact microbes	*In vitro*; pilot materials	Closest to commercialization	Regulatory harmonization	Sim et al., 2018; Díez-Pascual, 2020

* Early entries emphasize biogenic synthesis and antimicrobial evaluation, whereas later entries reflect application-level and translational trends and do not necessarily imply fully biogenic nanoparticle synthesis.

## 10 Future prospects and research directions

Despite substantial experimental progress, it is important to note that no biogenic nanoparticle-based antimicrobial has yet reached routine clinical use, and most reported benefits derive from laboratory and early preclinical studies. Looking ahead, biogenic nanoparticles (BNPs) may progress from experimental tools to rationally engineered “nano-antibiotics” for combating WHO-priority and ESKAPE pathogens. Achieving this will require tighter integration of green biosynthesis, computational design, and mechanistic microbiology to develop BNPs as precise anti-AMR agents rather than empirically tested adjuncts. In this context, artificial intelligence and machine learning offer a promising framework to guide BNP design, predict antimicrobial activity and resistance, and optimize combinations and pharmacokinetic performance against multidrug-resistant pathogens (Branda and Scarpa, 2024).

Recent advances demonstrate that BNPs synthesized from plants and microbes exhibit strong activity against MDR pathogens while maintaining superior biocompatibility and ecological safety compared with many chemically produced counterparts. Biogenic AgNPs, in particular, disrupt bacterial membranes, inhibit efflux systems, and synergize with conventional antibiotics, supporting their investigation as candidate nano-enabled antimicrobial systems, pending further validation (Singh et al., 2018). In parallel, AI- and machine-learning frameworks are emerging as powerful tools for predicting antimicrobial performance from nanoparticle size, shape, surface chemistry and capping biomolecules, enabling safe-by-design optimization. These models can guide the rational engineering of BNPs with enhanced bactericidal activity and reduced toxicity, paving the way for in silico–driven nanoantimicrobials tailored to overcome AMR (Mirzaei et al., 2021). Building such AI-guided design loops around biogenic platforms, rather than purely synthetic nanomaterials, should be a priority, as it aligns advanced computation with sustainable production routes.

A second strategic direction is the integration of multi-omics and systems biology to decode BNP–pathogen interactions at molecular resolution and to inform rational combination therapies. Recent work has shown that integrated transcriptomics, proteomics, metabolomics, and lipidomics can identify key bacterial pathways perturbed by antimicrobial nanomaterials, including oxidative stress responses, cell-envelope remodeling, DNA-damage repair, and metabolic bottlenecks (Wang Z. et al., 2025). In parallel, multi-omics frameworks are being used to map resistance determinants and adaptive responses in AMR pathogens (Tran and Dahlin, 2024). Applying such integrated omics systematically to BNP-treated MDR strains would allow researchers to pinpoint synergy nodes (e.g., specific stress-response regulators or membrane transporters) and to design BNP–antibiotic or BNP–host-directed combinations that are less prone to resistance. This approach also offers a route to identify biomarkers for BNP response, which could support future stratified or precision nanotherapies in the clinic. At the production level, biosynthetic pathway engineering and synthetic biology offer powerful opportunities to transform BNPs from heterogeneous laboratory products into tunable therapeutic platforms. Plant-, microbe-, and algae-based green synthesis is now well established for metal and metal-oxide nanoparticles, but most systems remain poorly optimized and strongly dependent on native metabolite profiles (Alsaiari et al., 2023).

Future work should focus on engineering microbial and plant chassis, through targeted manipulation of redox enzymes, metal transporters, and secondary metabolite pathways, to control nucleation rates, capping chemistry, and valence states, thereby narrowing size distributions and stabilizing antimicrobial surface motifs. Recent reviews on microbial nanoparticle production emphasize how physicochemical factors and metabolic fluxes shape NP morphology and catalytic/antimicrobial properties, underscoring the feasibility of pathway-level control (Bhatnagar and Aoyagi, 2025). Coupling such engineered biosynthetic platforms with real-time process analytics (e.g., inline spectroscopy and ML-based process control) could generate pharmaceutically compliant BNPs with batch-to-batch consistency suitable for drug development.

Therapeutically, hybrid nanosystems that integrate BNPs with polymers, peptides, or classical antibiotics are likely to dominate the next wave of anti-AMR applications. Antimicrobial peptide (AMP)-decorated silver nanoparticles have already demonstrated enhanced bactericidal and anti-biofilm activity against MDR pathogens compared with either component alone, leveraging multivalent binding, membrane disruption, and improved delivery of the peptide cargo (Xu et al., 2021). Similarly, synergistic systems combining metal nanoparticles with antibiotics or other adjuvants have been shown to lower minimum inhibitory concentrations, restore activity of legacy drugs, and overcome biofilm-associated tolerance (Ribeiro et al., 2022). Translating these concepts into fully biogenic or semi-biogenic constructs, where the core or shell is produced via green synthesis and conjugated to peptides, polysaccharides, or biodegradable polymers, could yield nanoformulations that are both potent against AMR pathogens and acceptable to regulators and patients from a safety and sustainability perspective.

Finally, the future of BNP-based anti-AMR strategies must be embedded in sustainable, circular bioeconomy frameworks. Recent work has highlighted the feasibility of synthesizing antimicrobial nanoparticles from agricultural residues, food-processing by-products, and other low-value biomass streams, effectively integrating nanoparticle production into broader biorefinery schemes (Cardoso et al., 2025b). Such models can reduce the environmental footprint and cost of BNP production while creating additional value chains in agriculture and aquaculture. At the same time, “safe-by-design” principles, combining ecotoxicological assessment, life–cycle analysis, and adaptive regulatory science, must be applied early, to ensure that BNP-based nanoantibiotics do not introduce new long-term ecological or resistance risks. Linking these sustainable production pipelines with global AMR surveillance data and horizon-scanning initiatives will allow prioritization of BNP candidates that target the most pressing resistant pathogens and fit within realistic manufacturing, regulatory, and stewardship frameworks (Chaturvedi and Ranjan, 2025). In General, the next decade of research should move from proof-of-concept demonstrations toward integrated platforms that combine AI-guided design, multi-omics-guided mechanistic insight, engineered biogenic production, hybrid therapeutic architectures, and sustainable biorefinery-based manufacturing. If pursued strategically and collaboratively, these directions could position biogenic nanoparticles not only as ecological curiosities of green chemistry but as credible, clinically relevant drug modalities against the evolving landscape of antimicrobial resistance.

## 11 Conclusion

This review demonstrates that biogenic nanoparticles offer a scientifically credible and environmentally sustainable path toward developing next-generation antimicrobial agents capable of addressing the accelerating global crisis of antimicrobial resistance. By harnessing the reducing and stabilizing potential of plant- and microbe-derived metabolites, BNPs achieve unique physicochemical properties, such as small size, natural surface coronas, and high reactivity, that collectively underpin their broad-spectrum antibacterial and antibiofilm activities. Evidence gathered across biological systems confirms that BNPs act through multiple, mutually reinforcing mechanisms, enabling potent inhibition of WHO-priority and ESKAPE pathogens and frequent synergy with conventional antibiotics. The central argument advanced in this review is that BNPs are not merely green alternatives to synthetic nanoparticles but constitute a mechanistically distinct class of nano-enabled antimicrobials with significant promise for clinical, industrial, and biotechnological use. Their increasing integration into wound dressings, drug delivery systems, biosensors, and food packaging highlight their translational relevance. However, the field still faces substantial barriers, including heterogeneity in green-synthesis protocols, limited mechanistic depth at the molecular level, scalability challenges, and insufficient long-term safety assessments. These limitations must be addressed to ensure that BNP-based technologies progress from experimental systems to reliable, reproducible, and clinically acceptable solutions. Ultimately, the synthesis, mechanisms, applications, and challenges outlined throughout this review converge to reinforce the overarching message: biogenic nanoparticles hold great potential as future nano-antibiotics and multifunctional antimicrobial platforms. Advancing this field requires interdisciplinary efforts integrating green chemistry, mechanistic microbiology, toxicology, and regulatory science. When these elements are aligned, BNPs could become a cornerstone of innovative, sustainable strategies to mitigate the global AMR threat.
